# Urban Housing and Hypertension Among Women in India: Comparing Slum and Non-Slum Contexts Using National Survey Data

**DOI:** 10.3390/ijerph22121817

**Published:** 2025-12-04

**Authors:** Uchita Vaid, Wanting Jiang

**Affiliations:** 1 Department of Design Studies, University of Wisconsin—Madison, Madison, WI 53705, USA; 2 Department of Consumer Science, University of Wisconsin—Madison, Madison, WI 53705, USA; wjiang96@wisc.edu

**Keywords:** housing quality, hypertension, slums, social determinants of health, India, women’s health, urban inequalities, low- and middle-income countries, national family health survey, health disparities

## Abstract

Housing conditions are increasingly recognized as critical determinants of non-communicable diseases; however, their influence on hypertension (HTN) risk remains underexplored in low- and middle-income countries. In urban India, structural disparities in housing are especially pronounced between slum and non-slum areas, making comparative analysis crucial for understanding context-specific health risks. This study examines the relationship between multidimensional housing conditions and HTN risk among women aged 18–49, drawing on data from 68,422 respondents in the fourth National Family Health Survey. A composite housing index was developed to capture six dimensions: structural quality, housing services access, indoor air quality, crowding, tenure security, and asset ownership. Survey-weighted logistic regressions were used to assess associations between housing conditions and HTN, controlling for key socio-demographic and health-related factors. We found that overall HTN prevalence was lower in slum households (11.6%) than in non-slum households (16.0%). Unexpectedly, slum households reported better structural durability and indoor air quality than non-slum households, suggesting incremental improvements in notified or tenure-secure slums. Better tenure security and asset ownership were found to be protective factors for HTN risk, while better structural quality was associated with higher HTN odds in non-slum areas. Crowding showed contrasting effects: in slums, higher crowding increased HTN risk, whereas in non-slums, lower crowding was associated with higher HTN. These findings highlight the context-dependent nature of housing-health links. Targeted interventions that address both physical infrastructure and broader living conditions can play a vital role in reducing urban hypertension disparities among women in India.

## 1. Introduction

This study examines the influence of multidimensional housing conditions on hypertension (HTN) among women living in urban slums in India. Urban slum residents, particularly women, often experience compounded social, economic, and environmental stressors that may heighten cardiovascular risk, yet research explicitly examining housing–hypertension links in these populations remains limited. By comparing women living in slum and non-slum environments, this study aims to illuminate how context-specific housing conditions shape hypertension risk in rapidly urbanizing low- and middle-income countries (LMICs).

Hypertension (HTN) is a major risk factor for a range of serious health conditions, including ischemic heart disease, stroke, cardiovascular disease, chronic kidney disease, and dementia [1]. Defined as systolic blood pressure (SBP) ≥ 140 mmHg or diastolic blood pressure (DBP) ≥ 90 mmHg, hypertension has become increasingly prevalent worldwide, with particularly sharp rises observed in low- and middle-income countries (LMICs) [2]. Extensive evidence documents the public health benefits of lowering blood pressure and preventing hypertension to reduce the burden of cardiovascular and other chronic diseases [3]. Given that HTN is the leading modifiable risk factor for cardiovascular disease and premature mortality in India and globally, identifying and addressing its risk factors remains a public health priority [4].

Well-established risk factors for HTN include metabolic and behavioral pathways such as body mass index, unhealthy diet, tobacco and alcohol use, and physical inactivity [5]. However, a growing body of research highlights that hypertension risk is also shaped by social determinants of health (SDoH), the conditions in which people are born, live, and work. For instance, socioeconomic factors such as educational attainment, income, and occupation have been linked to HTN risk [6,7]. A review conducted in high-income countries highlighted that environmental determinants such as material conditions, including housing and food access, also play important roles in hypertension risk [8].

The Healthy People 2030 framework classifies SDoH into five interrelated domains: economic stability, education access and quality, healthcare access and quality, neighborhood and built environment, and social and community context [9]. As part of the built environment domain, housing, in particular, has emerged as a critical yet underexplored determinant of cardiovascular health [10,11]. Multiple dimensions of housing, including affordability, stability, quality, and neighborhood characteristics, serve as pathways linking housing to health [12]. However, housing still remains an underexplored risk factor for HTN [13], especially in LMICs, where the burden of harsh living conditions can be further exacerbated.

Evidence from high-income economies shows that neighborhood-level conditions such as safety, crime rates, walkability, neighborhood socioeconomic status, homeownership, residential mobility, and foreclosure rates are associated with HTN risk [14,15]. Living in deprived neighborhoods has been consistently associated with elevated HTN risk. For example, U.S.-based studies found that residents of socioeconomically disadvantaged neighborhoods faced a higher risk of coronary heart disease, even after accounting for individual-level income, education, and occupation [16,17]. In addition to these neighborhood aspects, housing-level factors are also critical, which is the main focus of this study.

Housing-level characteristics, including stability, affordability, and quality, have been linked to HTN. Housing instability, defined using indicators such as difficulty paying rent, homelessness, temporary housing, frequent relocations, and insecure tenure, has been associated with higher HTN risk in both population and individual-level studies [18,19]. These associations are often patterned by gender, race, and class. For example, findings from the Coronary Artery Risk Development in Young Adults (CARDIA) study identified significantly elevated HTN risk among unstably housed women compared to stably housed white women but not for white men, black women, or black men [20]. Similar relationships have been noted for housing affordability, a closely related dimension of housing stability [21,22]. Such findings make a strong case for nuanced analyses of context-dependent housing-health links.

Poor housing quality is an important social determinant of health for both physical [23] and mental health conditions [24]. Housing quality factors such as thermal quality and indoor air quality [25], as well as exposure to harmful toxins [26], have been shown to be important risk factors for HTN. Other housing quality factors have also been theorized to be possible risk factors for HTN and cardiovascular health, such as overcrowding, durability, and structural quality, as well as access to water and sanitation infrastructure [27]. However, the impacts of these housing aspects on HTN need further empirical investigation.

In LMICs, particularly within urban slum settlements, poor housing conditions are compounded by social and economic marginalization, environmental hazards, and the persistent threat of eviction [28]. The United Nations defines a slum household as an urban dwelling in which residents lack at least one key conditions of adequate housing, namely structural durability, sufficient living space, safe and affordable water, adequate sanitation, or security of tenure protecting against forced eviction [29]. The term “slum” remains contentious within academic discourse, primarily due to its historically pejorative connotations that risk reinforcing stigmatizing perceptions of particular settlements and their inhabitants [30]. Nonetheless, this paper adopts the term “slum” to refer to the settlements under investigation, in alignment with its prevalent use by local authorities and international bodies such as the United Nations to classify communities sharing similar structural, spatial, and socioeconomic attributes. Our usage is purely descriptive and not intended to perpetuate negative stereotypes. Existing work provides a comprehensive rationale behind the use of this terminology [31].

The layered stressors in slum settlements have significant implications for cardiovascular health [32], yet research explicitly examining housing–hypertension relationships in slum settings remains limited despite the scale of urban slum populations worldwide [33]. Moreover, existing studies rarely compare slum and non-slum environments directly, leaving important questions about how housing risks manifest differently across these distinct contexts. Addressing this gap is essential for understanding the context-specific pathways through which housing influences hypertension in rapidly urbanizing LMICs such as India, particularly among women, who often experience disproportionate burdens of housing insecurity.

While slum housing in India is often characterized by precariousness and poor material conditions, recent research points to important heterogeneities within these environments. In many notified or long-established slum settlements, residents gradually invest in physical upgrades once a degree of tenure security is achieved, resulting in housing structures that may, in some respects, surpass those found in older or neglected non-slum areas [34]. This complexity underscores the need to move beyond binary understandings of “formal” versus “informal” housing and to examine how varied urban trajectories shape health outcomes. The present study contributes to this discussion by testing whether specific housing dimensions, including structural durability, service access, and crowding, are differentially associated with hypertension among women living in slum and non-slum contexts.

This study addresses these gaps by examining the association between multi-dimensional housing conditions and hypertension among women aged 18–49 years in urban India, using data from the National Family Health Survey (NFHS-4) across seven Indian states that explicitly distinguish between slum and non-slum areas. Specifically, we ask the following questions: (a) How do housing conditions and hypertension prevalence differ between women living in slum settlements and those in non-slum urban areas? (b) How are multidimensional housing conditions associated with hypertension risk in urban India, and do these associations vary between slum and non-slum settlements? By explicitly contrasting slum and non-slum environments, this study advances understanding of how urban housing inequalities shape women’s cardiovascular health in LMIC contexts.

The article is structured as follows: Section 2 details the methodology and sample selection process. Section 3 reports the main results for the research findings, highlighting the differences in housing conditions between urban slums and non-slum areas as well as associations between housing conditions and hypertension. Section 4 offers a discussion followed by the conclusion in Section 5.

## 2. Methods

### 2.1. Sample Selection

This study uses data from the 2015–2016 NFHS-4, conducted by the Ministry of Health and Family Welfare, Government of India. The survey employed a two-stage cluster random sampling design and collected nationally representative district-level data on socio-demographic characteristics, health indicators, and housing conditions through structured interviews with 723,875 eligible women aged 15–49 years. Notably, NFHS-4 expanded its scope to include clinical, anthropometric, and biochemical measurements, including blood pressure and blood glucose levels. Detailed survey methodologies, including sampling procedures, are available in the official NFHS-4 report [35]. Access to the individual-level NFHS dataset can be obtained upon request from the Demographic Health Survey (DHS) data repository [36].

NFHS-4 includes slum identification data from seven Indian states: Madhya Pradesh, Maharashtra, Delhi, Tamil Nadu, Uttar Pradesh, West Bengal, and Telangana. Given our research objective of comparing urban slum and non-slum areas, we relied on the NFHS-4 classification of slum versus non-slum clusters. The NFHS data include slum clusters in its sampling frame based on the identification of settlements by local Municipal Corporation Offices (35). They also include observations of the survey enumerators based on indicators such as density, housing quality, water access, and sanitation (36). A household is classified as a slum household in our analysis if it was identified as a slum by the local Municipal Corporation or by the survey enumerator. This approach provides a more holistic identification of slums, as it captures both the legal designation and the observed living conditions that reflect slum characteristics. Since slum classification was conducted only in eight Indian cities from seven states, our analysis is limited to data from these seven states. This restricted the sample to 68,422 eligible women.

**Figure 1:**
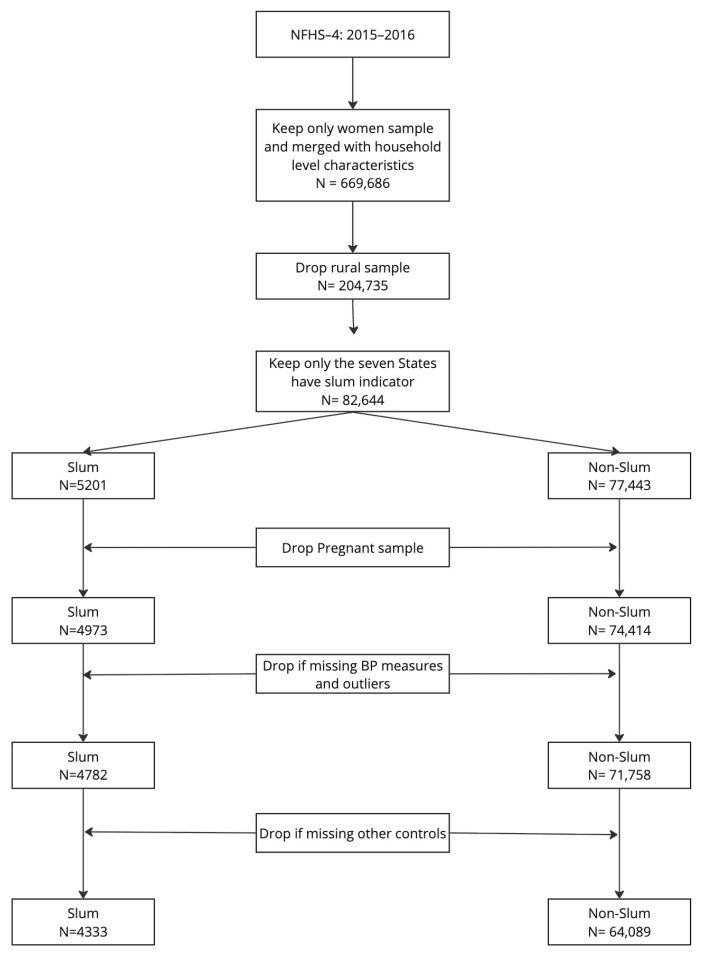
Data exclusion criteria and final sample sizes.

Blood pressure measurements were recorded for each eligible woman using an Omron Blood Pressure Monitor, with three readings taken at five-minute intervals. We excluded participants if all three systolic and diastolic blood pressure (SBP and DBP) readings were missing, as their hypertension status could not be determined. Additionally, pregnant women were excluded to avoid the confounding effects of pregnancy-associated hypertension [15]. Participants aged less than 18 years old and with missing data on housing variables or key demographic controls were also removed. After these exclusions, the final sample comprised 68,422 women, including 4333 residing in urban slum areas and 64,089 in urban non-slum areas (Figure 1).

### 2.2. Dependent, Explanatory Variables, and Controls

#### 2.2.1. Hypertension

The dependent variable in this study is a binary indicator of hypertension. Participants are classified as hypertensive if they meet one of the following criteria: 1. reported having been diagnosed with hypertension by a doctor, or 2. had average systolic and/or diastolic blood pressure (SBP and DBP) readings above established thresholds (SBP ≥ 140 mmHg or DBP ≥ 90 mmHg). This criterion ensures comprehensive identification of both diagnosed and undiagnosed hypertension cases [36].

#### 2.2.2. Housing Quality

Housing condition or quality is the key explanatory variable, conceptualized across six dimensions based on the Slum Severity Index [37] and the Living Conditions Diamond framework [38]. These six housing dimensions include (i) Durability and Structural Quality, (ii) Access to Housing Services and Infrastructure, (iii) Household Possessions, (iv) Indoor Air Quality, (v) Crowding, and (vi) Tenure Security.

The dimension of ‘Durability and Structural Quality’ includes aspects of building materials used for floors, walls, and roofs for each housing unit. The more permanent materials are rated as better housing conditions than temporary materials. ‘Access to Housing Services and Infrastructure’ covers access to and location of essential utilities like water, sanitation, and electricity. The third dimension of ‘Household Possessions’ measured through ownership of essential assets such as a mattress, bed, refrigerator, and washing machine, as a proxy for living standards. ‘Indoor Air Quality’ dimension is assessed using indicators such as cooking fuel type and ventilation, which impact airborne pollutants. ‘Crowding’ is defined by the number of individuals per room. Finally, ‘Tenure Security’ reflects legal property rights and ownership status, affecting housing stability and investment in home improvements. Collectively, these six dimensions provide a comprehensive assessment of housing conditions in relation to the health outcome of hypertension. See Supplementary Materials (File S1: Detailed overview of study variables) for a detailed description of items included in each housing dimension.

Indicators within each domain were scored on a three-point ordinal scale (0 = poor quality, 1 = acceptable quality, 2 = good quality). Raw indicator scores were aggregated to form subscale (domain) scores. To account for differences in the number of indicators and variability across domains, subscale scores were standardized using a z-score transformation (i.e., subtracting the sample mean and dividing by the standard deviation). This procedure centers each domain’s distribution at zero with a standard deviation of one, allowing scores to be interpreted relative to the sample mean. Negative values indicate domain quality below the sample average, whereas positive values reflect above-average quality. These standardized domain scores were then averaged and re-standardized to generate dimension-specific scores that are directly comparable across domains. In all cases, higher scores indicate better overall housing quality.

#### 2.2.3. Controls

Multiple control variables were included in our analysis, including demographic factors, health behaviors, and healthcare access, as well as comorbidities related to hypertension based on existing research evidence.

The demographic factors that have been previously associated with hypertension, including the respondent’s current age, education level, marital status, and wealth index [39], were included as controls in this study. The wealth index used in this study is the pre-constructed NFHS/DHS measure, which is generated using principal components analysis applied to a set of household assets, dwelling characteristics, and access-to-service indicators [40]. This index captures household living standards and is widely used in demographic and health research. Health-related behaviors associated with hypertension, such as tobacco usage [41] and alcohol usage [42], were also included as controls.

Furthermore, access to healthcare has also been associated with hypertension [43]. Healthcare access was conceptualized as a binary measure, defined as ‘limited access’ if a respondent reported a “big problem” in obtaining care due to financial constraints, transportation issues, distance to facilities, lack of provider availability, or needing permission to seek care. Otherwise, they were classified as having ‘adequate healthcare access’.

Lastly, comorbidities known to influence hypertension, including diabetes, asthma, thyroid disorders, heart disease, cancer, and abnormal body mass index (BMI), were included as controls [44]. We created a single binary comorbidity indicator, coded as 1 if the respondent reported at least one of these conditions or had an abnormal BMI, and 0 if none were present. Abnormal BMI was defined according to World Health Organization (WHO) guidelines, with underweight classified as BMI < 18.5 and overweight as BMI ≥ 25 [45].

A detailed overview of all variables used in this study, including their coding and categorizations, is provided in the Supplementary Materials File S1: Detailed overview of study variables.

### 2.3. Statistical Analysis

Descriptive statistics were used to examine the distribution of demographic characteristics, health behaviors, healthcare access, comorbidities, and housing conditions by urban slum vs. non-slum residence. Bivariate analyses were conducted to examine differences in the independent variables between slum and non-slum households. Pearson’s chi-square test was used for comparing categorical variables, independent-sample *t*-tests for normally distributed continuous variables, and the Mann–Whitney *U* test for the non-normally distributed housing condition variables.

To assess the relationship between housing conditions and hypertension, we estimated survey-weighted logistic regression models. A single model specification was applied to three samples: the full sample (Model 1), slum households only (Model 2), and non-slum households only (Model 3). The regression model is defined as follows:

(1) $$\mathrm{logit}\;\left(P\left(Y_{i}=1|{\mathrm{Slum}}_{i}=s\right)\right)=\beta_{0}^{\left(s\right)}+\sum_{k=1}^{6}\beta_{k}^{\left(s\right)}\;{\mathrm{HD}}_{ki}+\gamma^{{\left(s\right)}^{\prime }}D_{i}+\delta^{{\left(s\right)}^{\prime }}H_{i}+\varepsilon_{i},\;s\in \{0,1\}$$

$Y_{i}$ denotes the binary indicator for hypertension status (1 = hypertensive, 0 = non-hypertensive) for individual i. The parameter s indexes whether the model is estimated for individuals living in slum areas (s=1) or non-slum areas (s=0). For completeness, we additionally estimate the same specification for the full sample, for which the superscript $\left(s\right)$ represents the pooled estimates. Each of the six standardized housing dimensions, durability and quality of structure, access to housing infrastructure, indoor air quality, crowding, household possessions, and tenure security, is entered simultaneously as an explanatory variable. The vectors $D_{i}$ and $H_{i}$ represent demographic and health-related control variables, respectively. Coefficients $\beta_{k}^{\;(1)}$ and $\beta_{k}^{\;(0)}$ capture the effects of each housing dimension within the slum and non-slum populations, respectively, while $\beta_{k}$ represents the corresponding estimates in the full sample model.

To examine the incremental contribution of each set of predictors, we estimate the models in three sequential blocks: (1) demographic factors only, (2) demographic and health-related factors combined, and (3) demographic, health-related, and housing dimensions jointly. We used z-test statistics to assess whether the determinants of hypertension differ significantly between slum and non-slum areas. We also compared the groups by including an interaction term for residence type and housing conditions in the estimated models. However, the interpretations of interaction terms can be complicated due to unobserved heterogeneity across the compared groups, so we chose to present the z-test comparisons here as the two analyses did not produce differing results [46]. For these analyses, associations were considered statistically significant at *p* < 0.05, and results are presented as crude and adjusted odds ratios (AORs) with 95% confidence intervals. All analyses were conducted using Stata (Version 18.5, StataCorp, College Station, TX, USA).

### 2.4. Survey Design and Weighting

All analyses used survey-weighted logistic regression to account for the complex survey design of the NFHS-4. The NFHS-4 employs a stratified, two-stage cluster sampling design, with census enumeration blocks (CEBs) serving as the primary sampling units (PSUs) in urban areas and villages as PSUs in rural areas. Sampling weights were applied to adjust for unequal probabilities of selection and non-response at the household and individual levels, ensuring representativeness at the national and state levels. These weights reflect the inverse of the probability of selection for each respondent, adjusted for non-response, and normalized so that the weighted number of cases equals the total number of unweighted cases at the national level [36].

## 3. Results

To examine the research questions, we conducted a two-step analysis. First, we compared women living in slum settlements with those in non-slum urban areas on demographic characteristics, health aspects, housing conditions, and hypertension prevalence. Second, we modeled the associations between demographic factors, healthcare access, housing conditions, and hypertension risk, both for women in slums and non-slums, as well as the full sample. The following sections present the findings.

### 3.1. Differences in Demographics, Hypertension Prevalence, and Housing Conditions Between Urban Slum and Non-Slum Groups

Results from the bivariate comparisons indicate several significant differences in demographics and health aspects between women in urban slum and non-slum areas (Table 1). Women in slums reported significantly lower educational attainment (*p* < 0.001), with 37.1% having no formal education or only primary education, compared to 31.1% in non-slum areas. The marriage rates, currently married or formerly married, were slightly higher in slums (81.2%) than in non-slum households (80.1%), with *p* = 0.005.

Hypertension-related measures also showed significant differences. Women in non-slum areas had higher average systolic (114.12 mmHg vs. 112.90 mmHg) and diastolic (77.07 mmHg vs. 76.43 mmHg) blood pressure readings (*p* < 0.001). As for the prevalence of hypertension, 11.6% women were hypertensive in slum areas as compared to a significantly higher 16.0% in non-slum areas (*p* < 0.001). 64.5% of slum residents reported having access to healthcare, which was significantly different than 59.4% in non-slum areas. Tobacco use was significantly lower among slum residents (4.2% vs. 5.5%), whereas alcohol consumption did not significantly differ in the two groups (*p* = 0.877). No significant differences were found in the prevalence of comorbid conditions either (*p* = 0.323).

Housing conditions varied significantly between the two groups, although the direction of these differences varied for different housing dimensions (Table 2). Non-slum households reported better access to housing services and infrastructure, lower levels of crowding, more household possessions, and higher tenure security. In contrast, slum households reported better indoor air quality and higher durability and structural quality scores.

### 3.2. Association of Demographic Factors with Hypertension

Survey-weighted logistic regression models assessing the relationship between demographic factors and hypertension are presented in Table 3. In the full sample, several demographic characteristics were significantly associated with hypertension.

Age was positively correlated with hypertension, with each additional year increasing the odds of hypertension (AOR: 1.067, 95% CI [1.062–1.072]). Educational attainment also showed a significant relationship, where women with secondary or higher education had increased odds of hypertension compared to those with no formal education (AOR: 1.227, 95% CI [1.123–1.341]; AOR: 1.137, 95% CI [1.017–1.272]). Odds of having hypertension significantly increased with an increase in education levels. Wealth index followed a similar pattern, with women with higher wealth were more likely to be hypertensive (AOR: 1.048, 95% CI [1.014–1.083]. Marital status was also a significant predictor of hypertension, where unmarried women had reduced odds of being hypertensive in comparison to married women (AOR: 0.603, 95% CI [0.530–0.686]).

These associations remained largely consistent in the urban non-slum sample (Table 3; Columns 5–6), where significant variables, effect sizes, and directions remain consistent with the full sample. However, in the urban slum sample (Columns 3–4), education, marital status, and wealth index were not significantly associated with hypertension.

### 3.3. Association of Health Access and Comorbidities with Hypertension

Block 2 of the regression models incorporated healthcare access, tobacco and alcohol use, and presence of comorbidities as hypertension determinants, along with variables in block 1 (Table 4). The results show that in the full sample, women who reported no barriers to healthcare access had higher odds of hypertension (AOR: 1.174, 95% CI [1.101–1.252]). This association remained significant in both urban slum and non-slum subsamples but was stronger among slum residents (AOR: 1.763, 95% CI [1.260–2.467], *p* < 0.01). This finding indicates women with better healthcare access are more likely to report hypertension, likely due to diagnostic access. This effect is significantly stronger in slums, where people without access are even more likely not to be diagnosed.

Additionally, the absence of comorbid conditions was associated with lower hypertension risk in both the full sample (AOR: 0.725, 95% CI [0.680–0.772]) and the urban slum and non-slum subsamples (AOR: 0.457, 95% CI [0.320–0.653]; AOR: 0.740, 95% CI [0.694–0.789]), with a stronger effect in non-slum residents (z = −2.607, *p* < 0.01). This suggests that women without diagnosed co-existing illnesses such as diabetes, asthma, thyroid disorders, heart disease, cancer, or abnormal BMI had lower odds of hypertension. The stronger negative effect in slums suggests that co-existing illnesses are a key driver of hypertension in low-resource contexts.

Overall, results from block 2 indicate that socio-demographic factors such as education and wealth are inconsistent predictors once health aspects are included. Between-group differences in slums and non-slums are modest, suggesting that the basic demographic and health predictors operate similarly across housing contexts, except for health access and comorbidity, with more pronounced influence in slum contexts.

### 3.4. Association of Housing Conditions with Hypertension

**Figure 2:**
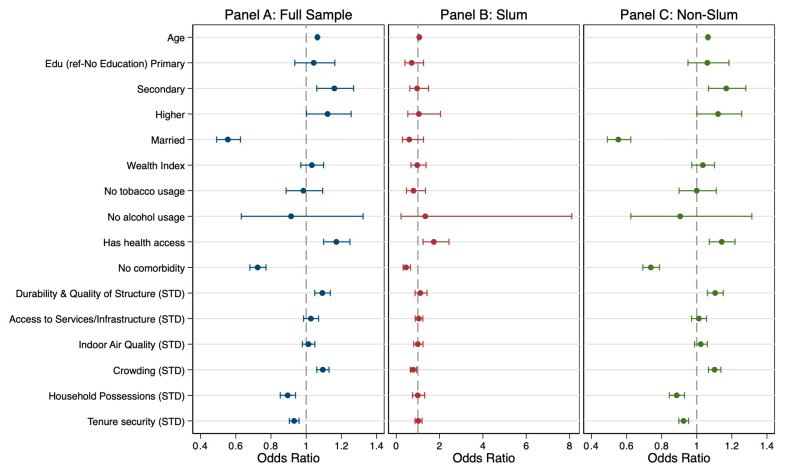
Adjusted odds ratios for hypertension in women in the full sample, slum, and non-slum groups for Block 3, including housing conditions as risk factors.

Table 5 presents findings from block 3 of the regression models, which incorporated housing conditions after controlling for demographic and health-related factors. Several housing dimensions were significantly associated with hypertension in the full sample, and a few of these association patterns varied between slum and non-slum contexts. These associations are visually summarized in Figure 2, which presents adjusted odds ratios for both subsamples.

In the full sample, the Durability and Quality of Structure variable was positively associated with hypertension (AOR: 1.084, 95% CI [1.041–1.128]), indicating that better structural quality corresponded with greater odds of hypertension. This pattern remained significant in the non-slum sample (AOR: 1.096, 95% CI [1.053–1.142]) but was not observed in the slum sample. This pattern suggests that in better-built environments, higher socioeconomic status and improved access to health services may coincide with greater detection of hypertension, potentially reflecting diagnostic or reporting differences rather than a direct physiological effect of housing quality.

Crowding showed opposing relationships across housing contexts. In the full sample, lower crowding levels were linked to higher hypertension odds (AOR: 1.096, 95% CI [1.061–1.132]), a pattern also present in the urban non-slum sample. In contrast, among slum residents, the association was reversed: greater crowding was linked to lower hypertension risk (AOR = 0.782, 95% CI [0.650–0.940]). The difference in crowding effects between slum and non-slum contexts was statistically significant (z = –3.598, *p* < 0.001), suggesting that density operates differently depending on spatial and social configurations of housing. In slum settings, higher crowding may be more indicative of strong social ties or household sharing arrangements rather than environmental stress, whereas in non-slum areas, it may reflect space constraints and psychosocial strain.

Household Possessions were inversely associated with hypertension in both the full (AOR = 0.895, 95% CI [0.852–0.940]) and non-slum samples (AOR = 0.886, 95% CI [0.844–0.931]), implying that greater material asset ownership, an indicator of household wealth and comfort, was protective against hypertension. The association was not statistically significant among slum residents, suggesting that material assets may not confer similar health benefits where overall living conditions and other stressors remain high.

Tenure Security also demonstrated a protective association with hypertension in the full sample (AOR = 0.930, 95% CI [0.902–0.958]) and in the non-slum subsample (AOR = 0.922, 95% CI [0.895–0.951]). These findings indicate that secure housing tenure, measured by housing ownership in this study, is linked to lower hypertension risk, potentially through reduced chronic stress and enhanced residential stability. However, this association was not significant in slum areas, where the protective effects of tenure security may be manifesting in other forms than housing ownership. By contrast, Indoor Air Quality and Access to Housing Services and Infrastructure were not significantly associated with hypertension in any of the models. The lack of association suggests that within this urban sample, variability in these dimensions may be insufficient to meaningfully influence hypertension risk once demographic, health factors, and other housing dimensions are accounted for.

## 4. Discussion

This study underscores the significance of housing conditions as determinants of women’s hypertension in urban India, while highlighting that their effects differ between slum and non-slum contexts. Using NFHS-4 data from over 68,000 women, we found that hypertension prevalence is higher in non-slum households, yet the mechanisms linking housing and health diverge across housing settings. These differences in urban and non-urban contexts emphasize that urban housing-health relationships cannot be understood in aggregate terms alone; comparative analyses are essential to uncover how material and social contexts shape risk differently.

### 4.1. Housing Disparities in Urban Slums and Non-Slum Contexts

Our findings reveal housing disparities between slum and non-slum households that are consistent with established research, yet they also uncover patterns that challenge dominant narratives about informal settlements and their associated health risks. Interpreting these findings in the context of existing literature helps clarify how urban housing environments shape women’s lived experiences and inform pathways relevant to hypertension risk.

Consistent with longstanding evidence, slum households had substantially lower access to basic infrastructure, including water, sanitation, and electricity. Prior research attributes these deficits to political neglect, weak municipal service delivery, and regulatory barriers that historically exclude informal settlements from urban planning systems [41,42]. These persistent infrastructural gaps reinforce material deprivation and increase the daily burdens of managing health and hygiene, especially for women who disproportionately perform household labor. This alignment with existing scholarship reaffirms that infrastructural inequality remains a core dimension of slum disadvantage.

Crowding emerged as another important dimension with nuanced implications. High levels of crowding in slum households reinforce existing evidence that dense spatial conditions amplify psychosocial stress, particularly when combined with insufficient basic services [47]. Because crowding is theorized to influence cardiovascular outcomes through chronic stress pathways, its prominence in slum settings underscores a plausible mechanism linking the physical environment to hypertension risk.

At the same time, some findings diverge from common assumptions and merit deeper interpretation. Despite widespread perceptions that slum housing is uniformly substandard, we found that slum households exhibited better structural durability and indoor air quality than many non-slum households. This unexpected pattern contributes to a growing body of work demonstrating that slum housing evolves over time as residents engage in incremental upgrading once tenure becomes more secure [48,49]. Conversely, some non-slum areas, particularly older or peri-urban neighborhoods, may suffer from aging housing stock and weak municipal investment, reflecting a different form of vulnerability that is not rooted in informality but in institutional neglect [50,51].

Finally, lower levels of household asset ownership in slum areas point to the broader economic constraints that continue to shape urban inequality. Although structural quality may improve through long-term investment, asset-based deprivation signals persistent economic precarity and constrained adaptive capacity, both of which are associated with poorer long-term health outcomes [52,53]. This distinction highlights the multidimensional nature of slum disadvantage: improvements in built structures do not necessarily translate into improvements in economic well-being or reductions in stress-related health risks.

### 4.2. Divergent Housing-Health Pathways in Slum and Non-Slum Areas

We found that hypertension prevalence among women was 11.6% in slum areas and 16.0% in non-slum areas, underscoring both the overall burden and the contextual variation of hypertension in urban settings. This variation emphasizes the importance of comparing across settlement types to better understand urban health inequalities.

Several demographic characteristics were significant risk factors for hypertension. In both slum and non-slum areas, women with better access to healthcare were more likely to be classified as hypertensive. In this study, hypertension status was defined as either (a) having a medical diagnosis of hypertension or (b) having systolic or diastolic blood pressure readings above established clinical thresholds. Thus, the measure captures both true physiological prevalence and clinically detected cases. The higher likelihood of hypertension among women with better healthcare access does not necessarily indicate greater biological risk. Instead, it likely reflects increased opportunities for detection, as those with fewer access barriers are more likely to have their blood pressure assessed in clinical settings [43]. This distinction between true prevalence and diagnosed prevalence is well-documented in population health research [54]. Our findings extend this line of work by showing how healthcare access differentially shapes observed hypertension rates across settlement contexts. Future studies should also consider treatment pathways and continuity of care, since diagnosis represents only the first step in disease management.

Women without comorbid conditions were less likely to report hypertension, a pattern consistent across both settlement types. This finding aligns with previous research linking multimorbidity and elevated hypertension risk [55,56,57,58]. Our results confirm that multimorbidity remains a central urban health concern, reinforcing the need for integrated care approaches, particularly in resource-constrained contexts.

Housing-related predictors displayed complex, context-dependent associations. Housing durability and structural quality were positively associated with hypertension in the full sample, suggesting that households with higher structural quality, which is often correlated with greater wealth, may face lifestyle-related risks such as higher consumption of calorie-dense foods, reduced physical activity, or occupational stress [59,60]. Importantly, this association persisted in non-slum households but not in slum households. By contrast, this relationship was absent in slum settings, where lower wealth and more physically active lifestyles may offset such risks. This divergence highlights that “better” material housing conditions do not uniformly equate to better health outcomes; they must be interpreted within the socioeconomic and behavioral contexts in which they occur [61,62].

Tenure security and asset ownership were protective factors against hypertension in non-slum settings, likely through reduced psychosocial stress and enhanced perceived stability [63,64]. Greater asset ownership may also signal higher disposable income, further buffering cardiovascular risk [14]. However, these associations were not significant in slum settings. This suggests that tenure security and possessions can operate differently in informal settlements, where de facto ownership and community organization often shape perceived security, in addition to legal housing ownership [65,66]. Our findings therefore support previous calls for multidimensional measures of tenure security that include length of occupation, settlement size, and community cohesion, to better assess health consequences of tenure security in slums [67].

Household crowding also displayed distinct, context-dependent associations. In non-slum areas, lower crowding was unexpectedly associated with higher hypertension odds, while in slums, higher crowding was associated with a lower risk. This divergence highlights the importance of contextualizing household density within cultural and environmental frameworks. For instance, in many collectivist societies, shared living spaces may be culturally normative and not perceived as crowded or stressful, and thus lower per-room occupancy may not be as significant a health advantage [68,69]. In slums, crowding can intensify competition for scarce resources such as water, sanitation, and space, exacerbating psychosocial stress and consequently hypertension risk [64,65].

Together, these findings provide strong evidence that housing conditions, alongside demographic factors, are significant determinants of hypertension in urban India. Crucially, the associations vary across slum and non-slum settlements, highlighting the need to examine context-specific mechanisms to understand the differences in structural, economic, and psychosocial stressors within such settings. These results call for more health-focused housing policies that go beyond infrastructure to address the lived housing experiences of urban women, particularly in resource-limited slum environments.

### 4.3. Limitations

This study has several limitations that should be considered when interpreting the findings. First, the analysis is based on cross-sectional data from NFHS-4, the only available wave that includes both blood pressure measurements and slum indicators. This restricts our ability to examine temporal or causal relationships between housing conditions and hypertension. Without longitudinal data, it is difficult to determine whether certain housing exposures preceded the onset of hypertension or were consequences of other socioeconomic shifts. Future studies would benefit from panel data or prospective cohort designs that allow for tracking health and housing trajectories over time.

Second, while the housing index developed in this study incorporates multiple dimensions, it is still limited to household-level indicators. It does not capture broader contextual factors such as neighborhood conditions, environmental exposures such as noise, pollution, or the social cohesion of the local environment. These neighborhood-level characteristics can have independent and interacting effects on cardiovascular health but remain unmeasured here. For instance, the presence of nearby healthcare facilities, walkability, or perceived safety might significantly influence both stress levels and access to preventive care [70]. Incorporating neighborhood-level variables would enhance the contextualization of the housing-health relationship.

Lastly, due to sample design constraints, the analysis does not fully account for regional or state-level variation in urban housing and health systems. India’s vast urban landscape is heterogeneous, and factors such as local governance, housing policy, and public health infrastructure vary widely across states. Incorporating state- or city-level analyses would help uncover these nuances and support more geographically targeted interventions.

### 4.4. Policy Implications

The findings from this study carry several important implications for urban health policy in India, particularly in the context of rapidly expanding urban populations and entrenched intra-urban inequalities. First, they underscore the need to recognize housing as a critical and actionable determinant of non-communicable diseases such as hypertension. Current public health frameworks often emphasize behavioral and biomedical risk factors but pay comparatively limited attention to the material and psychosocial conditions of housing that shape everyday exposures to stress, environmental hazards, and barriers to health-promoting behaviors.

Health-focused housing policies also need to be better integrated with national hypertension control initiatives, such as the India Hypertension Control Initiative (IHCI), which has made important strides in standardizing treatment protocols, training providers, and improving medication access [71]. Our findings suggest that incorporating housing assessments, both objective and subjective, into routine public health monitoring could enhance the effectiveness of such initiatives.

Our study highlights how the same housing attributes, such as structural quality, tenure security, and crowding, can operate differently across slum and non-slum settings. This suggests that one-size-fits-all housing or health interventions may be inadequate or even counterproductive. Instead, context-sensitive approaches that reflect the heterogeneity of urban living conditions are essential. For instance, infrastructure improvements may yield greater health benefits in slum contexts where basic services are lacking, while interventions focused on reducing sedentary lifestyles or dietary risks may be more pertinent in non-slum areas where structural conditions are relatively better but other lifestyle risk factors are more prevalent.

Finally, housing policy needs to be viewed not only through the lens of shelter provision but also as a lever for promoting health equity. The role of tenure security is particularly salient. Policies that enhance legal recognition, prevent forced evictions, and support community-led upgrading initiatives can reduce chronic stress and improve psychosocial well-being. Secure housing can also promote economic investment in dwellings, which may have downstream benefits for both physical and mental health. Yet, legal security alone may not be sufficient; perceived tenure stability and residents’ lived experience of safety and control over their environment are equally important.

In summary, integrating housing and public health policy is crucial for addressing the growing burden of hypertension among urban women in India. Future strategies should aim not only to improve physical infrastructure but also to enhance stability, dignity, and livability, especially for populations in slum environments.

## 5. Conclusions

This study demonstrates that housing conditions influence women’s hypertension risk in urban India, but the nature and strength of these associations vary substantially between slum and non-slum environments. By explicitly comparing these settings, the analysis shows that similar housing attributes, such as structural quality, crowding, and tenure security, do not operate uniformly across urban contexts. This reinforces that housing-health relationships cannot be generalized without attention to the social, economic, and infrastructural environments in which they occur. These findings contribute to ongoing debates about the heterogeneity of urban disadvantage.

Evidence that some slum households have achieved higher structural quality than certain non-slum dwellings challenges conventional assumptions and highlights how incremental upgrading, tenure experiences, and urban governance shape health-relevant exposures in different ways. At the same time, persistent deficits in basic services and higher crowding in slum areas underscore the need for targeted investment in essential infrastructure and stress-reducing living conditions. Overall, the study advances the field by identifying context-specific pathways linking housing and hypertension and by emphasizing the necessity of differentiated urban health strategies. As India continues to urbanize, integrating housing quality, tenure stability, and lived residential experiences into public health planning will be essential for reducing cardiovascular risk and promoting health equity among urban women.

## Figures and Tables

**Table 1:** Demographic and health aspect comparisons of women in slum and urban non-slum populations of seven Indian states, NFHS-4, 2015–2016.

	Residence Type		
	Slum	Non-Slum	Bivariate Test *
	Share of Observations (%)	Share of Observations (%)	
N	4333 (6.3%)	64,089 (93.7%)	
Age Mean (SD)	31.774 (8.964)	31.973 (9.079)	0.162
Median	31	31	
Min–Max	18–49	18–49	
Education			
No education	1050 (24.2%)	12,413 (19.4%)	<0.001
Primary	558 (12.9%)	7514 (11.7%)	
Secondary	2043 (47.2%)	28,702 (44.8%)	
Higher	682 (15.7%)	15,460 (24.1%)	
Marital status			
Married	3269 (75.4%)	48,291 (75.4%)	0.005
Never Married	811 (18.7%)	12,695 (19.8%)	
Formerly Married	253 (5.9%)	3103 (4.8%)	
Wealth Index Mean (SD)	4.062 (0.921)	4.039 (1.077)	0.156
Median	4.0	4.0	
Min–Max	1–5	1–5	
State			
Madhya Pradesh	1067 (24.6%)	14,775 (23.0%)	<0.001
Maharashtra	926 (21.4%)	8313 (13.0%)	
Delhi	230 (5.3%)	3829 (6.0%)	
Tamil Nadu	502 (11.6%)	10,706 (16.7%)	
Uttar Pradesh	766 (17.7%)	20,999 (32.8%)	
West Bengal	402 (9.3%)	3705 (5.8%)	
Telangana	440 (10.1%)	1762 (2.7%)	
Hypertension Prevalence			
Has Hypertension	501 (11.6%)	10,255 (16.0%)	<0.001
Average Systolic Mean (SD)	112.897 (13.661)	114.123 (13.339)	<0.001
Median	111.67	112.67	
Min–Max	69–169	66–169	
Average Diastolic Mean (SD)	76.426 (8.989)	77.070 (8.698)	<0.001
Median	76.67	77.0	
Min–Max	46.0–133.5	42.3–146.0	
Comorbidity			
Yes	2191 (50.6%)	31,910 (49.8%)	0.323
Healthcare Access			
No Health Access	2795 (64.5%)	38,084 (59.4%)	<0.001
Tobacco Usage			
Yes	182 (4.2%)	3511 (5.5%)	<0.001
Alcohol Usage			
Yes	21 (0.5%)	300 (0.5%)	0.877

Notes: * Independent *t*-tests were conducted for continuous variables, and Pearson’s chi-square tests were used for categorical variables to assess differences between the two groups.

**Table 2:** Housing conditions across six housing dimensions in urban slums and non-slum population of seven Indian states, NFHS-4, 2015–2016.

Housing Conditions	Residence Type		
	Slum Mean (SD); Median	Non-Slum Mean (SD); Median	*p*-Value
Durability & Quality of Structure	0.256 (0.745); 0.476	−0.017 (1.013); 0.477	<0.001
Access to Housing Services/Infrastructure	−0.084 (0.947); 0.247	0.006 (1.003); 0.251	<0.001
Indoor Air Quality	0.119 (0.787); 0.721	−0.008 (1.012); 0.721	<0.001
Crowding	−0.171 (0.944); −0.820	0.012 (1.003); −0.820	<0.001
Household Possessions	−0.079 (0.977); −0.217	0.005 (1.001); −0.217	<0.001
Tenure security	−0.114 (1.063); 0.555	0.008 (0.995); 0.555	<0.001

Notes: Each housing dimension score is standardized, and higher values indicate better housing conditions. Mann–Whitney *U* tests assess differences between groups to account for non-normal distributions.

**Table 3:** Adjusted and crude odds ratios for hypertension in women in the full sample, slum group, and non-slum group for block 1, including demographic risk factors.

	Full Sample		Slum		Non-Slum		z-Test
	Crude Odds Ratio	Adjusted Odds Ratio	Crude Odds Ratio	Adjusted Odds Ratio	Crude Odds Ratio	Adjusted Odds Ratio	
Age							
Odds Ratio [SE]	1.073 *** [0.002]	1.067 *** [0.002]	1.068 *** [0.010]	1.062 *** [0.012]	1.073 *** [0.002]	1.067 *** [0.002]	−0.420
(95% CI)	(1.069–1.077)	(1.062–1.072)	(1.048–1.087)	(1.039–1.085)	(1.069–1.077)	(1.062–1.072)	
Education (ref-No Education)							
Primary							
Odds Ratio [SE]	1.125 ** [0.051]	1.087 [0.060]	0.880 [0.219]	0.762 [0.218]	1.147 ** [0.062]	1.108 [0.062]	−1.286
(95% CI)	(1.029–1.231)	(0.975–1.212)	(0.539–1.434)	(0.435–1.334)	(1.047–1.256)	(0.993–1.237)	
Secondary							
Odds Ratio [SE]	1.084 ** [0.034)	1.227 *** [0.056]	0.909 [0.149]	0.984 [0.209]	1.098 ** [0.034]	1.239 *** [0.057]	−1.061
(95% CI)	(1.020–1.152)	(1.123–1.341)	(0.658–1.254)	(0.650–1.491)	(1.032–1.167)	(1.132–1.355)	
Higher							
Odds Ratio [SE]	0.702 *** [0.027]	1.137 * [0.065]	0.630 [0.171]	0.904 [0.296]	0.695 *** [0.027]	1.137 * [0.065]	−0.688
(95% CI)	(0.651–0.757)	(1.017–1.272)	(0.370–1.073)	(0.476–1.717)	(0.645–0.749)	(1.015–1.272)	
Marital Status (ref- Married)							
Never Married							
Odds Ratio [SE]	0.269 *** [0.015]	0.603 *** [0.040]	0.350 ** [0.118]	0.725 [0.269]	0.264 *** [0.015]	0.598 *** [0.039]	0.510
(95% CI)	(0.240–0.300)	(0.530–0.686)	(0.181–0.679)	(0.350–1.500)	(0.236–0.295)	(0.527–0.680)	
Formerly Married							
Odds Ratio [SE]	1.361 *** [0.086]	0.884 [0.058]	1.609 [0.442]	0.983 [0.290]	1.354 *** [0.087]	0.882 [0.059]	0.358
(95% CI)	(1.202–1.540)	(0.778–1.006)	(0.939–2.758)	(0.552–1.752)	(1.193–1.536)	(0.774–1.006)	
Wealth Index							
Odds Ratio [SE]	1.089 *** [0.016]	1.048 ** [0.018]	1.022 [0.092]	1.004 [0.105]	1.086 *** [0.015]	1.047 ** [0.015]	−0.394
(95% CI)	(1.059–1.120)	(1.014–1.083)	(0.856–1.221)	(0.818–1.232)	(1.056–1.117)	(1.013–1.082)	
Intercept							
Odds Ratio [SE]		0.017 *** [0.002]		0.019 *** [0.011]		0.017 *** [0.002]	
(95% CI)		(0.014–0.021)		(0.007–0.058)		(0.014–0.021)	
N		68,422		4333		64,089	

Note: Crude ORs represent unadjusted bivariate associations calculated from cross-tabulations, while adjusted ORs are derived from multivariate logistic regression models controlling for socio-demographic and health-related covariates. Column (7) reports the *z*-statistics testing whether the coefficients of interest differ significantly between the slum and non-slum samples. * Significant at *p* < 0.05; ** significant at *p* < 0.01; *** significant at *p* < 0.001.

**Table 4:** Adjusted and crude odds ratios for hypertension in women in the full sample, slum group, and non-slum group for block 2, including health behaviors, healthcare access, and comorbidity status as risk factors.

	Full Sample		Slum		Non-Slum		z-Test
	Crude Odds Ratio	Adjusted Odds Ratio	Crude Odds Ratio	Adjusted Odds Ratio	Crude Odds Ratio	Adjusted Odds Ratio	
Age							
Odds Ratio [SE]	1.073 *** [0.002]	1.064 *** [0.002]	1.068 *** [0.010]	1.057 *** [0.012]	1.073 *** [0.002]	1.064 *** [0.002]	−0.553
(95% CI)	(1.069–1.077)	(1.060–1.069)	(1.048–1.087)	(1.033–1.082)	(1.069–1.077)	(1.060–1.069)	
Education (ref-No Education)							
Primary							
Odds Ratio [SE]	1.125 ** [0.051]	1.076 [0.060]	0.880 [0.219]	0.705 [0.206]	1.147 ** [0.053]	1.097 [0.062]	−1.490
(95% CI)	(1.029–1.231)	(0.965–1.200)	(0.539–1.434)	(0.398–1.248)	(1.047–1.256)	(0.983–1.225)	
Secondary							
Odds Ratio [SE]	1.084 ** [0.034]	1.207 *** [0.055]	0.909 [0.149]	0.953 [0.207]	1.098 ** [0.034]	1.217 *** [0.056]	−1.100
(95% CI)	(1.020–1.152)	(1.104–1.320)	(0.658–1.254)	(0.623–1.459)	(1.032–1.167)	(1.112–1.332)	
Higher							
Odds Ratio [SE]	0.702 *** [0.027]	1.112 [0.064]	0.630 [0.171]	0.888 [0.296]	0.695 *** [0.027]	1.111 [0.064]	−0.663
(95% CI)	(0.651–0.757)	(0.994–1.245)	(0.370–1.073)	(0.462–1.708)	(0.645–0.749)	(0.992–1.245)	
Marital Status (ref- Married)							
Never Married							
Odds Ratio [SE]	0.269 *** [0.015]	0.610 *** [0.040]	0.350 ** [0.118]	0.739 [0.269]	0.264 *** [0.015]	0.605 *** [0.040]	0.544
(95% CI)	(0.240–0.300)	(0.536–0.695)	(0.181–0.679)	(0.363–1.507)	(0.236–0.295)	(0.532–0.688)	
Formerly Married							
Odds Ratio [SE]	1.361 *** [0.086]	0.894 [0.059]	1.609 [0.442]	0.897 [0.282]	1.354 *** [0.087]	0.895 [0.060]	0.007
(95% CI)	(1.202–1.540)	(0.786–1.017)	(0.939–2.758)	(0.485–1.661)	(1.193–1.536)	(0.785–1.021)	
Wealth Index							
Odds Ratio [SE]	1.089*** [0.016]	1.024 [0.018]	1.022 [0.092]	0.956 [0.102]	1.086 *** [0.015]	1.025 [0.018]	−0.641
(95% CI)	(1.059–1.120)	(0.991–1.060)	(0.856–1.221)	(0.776–1.179)	(1.056–1.117)	(0.991–1.060)	
No tobacco usage							
Odds Ratio [SE]	0.913 [0.046]	1.007 [0.055]	0.680 [0.167]	0.823 [0.225]	0.930 [0.047]	1.024 [0.056]	−0.783
(95% CI)	(0.827–1.007)	(0.906–1.120)	(0.420–1.100)	(0.482–1.407)	(0.842–1.028)	(0.920–1.140)	
No alcohol usage							
Odds Ratio [SE]	0.773 [0.146]	0.854 [0.158]	0.818 [0.862]	1.352 [1.244]	0.764 [0.143]	0.841 [0.158]	0.505
(95% CI)	(0.534–1.118)	(0.593–1.228)	(0.104–6.450)	(0.223–8.205)	(0.528–1.103)	(0.583–1.215)	
Has health access							
Odds Ratio [SE]	1.192 *** [0.037]	1.174 *** [0.038]	1.655 ** [0.273]	1.763 *** [0.302]	1.163 *** [0.037]	1.144 *** [0.038]	2.480 *
(95% CI)	(1.121–1.267)	(1.101–1.252)	(1.199–2.286)	(1.260–2.467)	(1.094–1.238)	(1.072–1.221)	
No comorbidity							
Odds Ratio [SE]	0.598 *** [0.019]	0.725 *** [0.024]	0.417 *** [0.075]	0.457 *** [0.083]	0.607 *** [0.019]	0.740 *** [0.024]	−2.607 **
(95% CI)	(0.562–0.637)	(0.680–0.772)	(0.293–0.592)	(0.320–0.653)	(0.571–0.647)	(0.694–0.789)	
Intercept							
Odds Ratio [SE]		0.026 *** [0.005]		0.026 *** [0.027]		0.026 *** [0.006]	
(95% CI)		(0.017–0.039)		(0.003–0.196)		(0.017–0.040)	
N		68,422		4333		64,089	

Note: Crude ORs represent unadjusted bivariate associations calculated from cross-tabulations, while adjusted ORs are derived from multivariate logistic regression models controlling for socio-demographic and health-related covariates. Column (7) reports the *z*-statistics testing whether the coefficients of interest differ significantly between the slum and non-slum samples. * Significant at *p* < 0.05; ** significant at *p* < 0.01; *** significant at *p* < 0.001.

**Table 5:** Adjusted and crude odds ratios for hypertension in women in the full sample, slum group, and non-slum group for block 3, including housing conditions as risk factors.

	Full Sample		Slum		Non-Slum		z-Test
	Crude Odds Ratio	Adjusted Odds Ratio	Crude Odds Ratio	Adjusted Odds Ratio	Crude Odds Ratio	Adjusted Odds Ratio	
Age							
Odds Ratio [SE]	1.073 *** [0.002]	1.063 *** [0.002]	1.068 *** [0.010]	1.061 *** [0.013]	1.073 *** [0.002]	1.063 *** [0.002]	−0.151
(95% CI)	(1.069–1.077)	(1.058–1.068)	(1.048–1.087)	(1.036–1.086)	(1.069–1.077)	(1.058–1.068)	
Education (ref-No Education)							
Primary							
Odds Ratio [SE]	1.125 ** [0.051]	1.047 [0.058]	0.880 [0.219]	0.717 [0.210]	1.147 ** [0.053]	1.064 [0.060]	−1.324
(95% CI)	(1.029–1.231)	(0.939–1.168)	(0.539–1.434)	(0.404–1.273)	(1.047–1.256)	(0.953–1.188)	
Secondary							
Odds Ratio [SE]	1.084 ** [0.034]	1.163 ** [0.053]	0.909 [0.149]	0.987 [0.220]	1.098 ** [0.034]	1.169 *** [0.054]	−0.743
(95% CI)	(1.020–1.152)	(1.063–1.273)	(0.658–1.254)	(0.638–1.527)	(1.032–1.167)	(1.067–1.281)	
Higher							
Odds Ratio [SE]	0.702 *** [0.027]	1.078 [0.063]	0.630 [0.171]	0.963 [0.330]	0.695 *** [0.027]	1.079 [0.063]	−0.325
(95% CI)	(0.651–0.757)	(0.962–1.209)	(0.370–1.073)	(0.492–1.886)	(0.645–0.749)	(0.961–1.210)	
Marital Status (ref-Married)							
Never Married							
Odds Ratio [SE]	0.269 *** [0.015]	0.611 *** [0.040]	0.350 ** [0.118]	0.739 [0.272]	0.264 *** [0.015]	0.605 *** [0.040]	0.533
(95% CI)	(0.240–0.300)	(0.536–0.695)	(0.181–0.679)	(0.359–1.520)	(0.236–0.295)	(0.532–0.688)	
Formerly Married							
Odds Ratio [SE]	1.361 *** [0.086]	0.868 * [0.058]	1.609 [0.442]	0.958 [0.298]	1.354 *** [0.087]	0.868 * [0.058]	0.310
(95% CI)	(1.202–1.540)	(0.763–0.989)	(0.939–2.758)	(0.521–1.761)	(1.193–1.536)	(0.761–0.990)	
Wealth Index							
Odds Ratio [SE]	1.089 *** [0.016]	1.034 [0.033]	1.022 [0.092]	0.965 [0.172]	1.086 *** [0.015]	1.037 [0.034]	−0.396
(95% CI)	(1.059–1.120)	(0.970–1.101)	(0.856–1.221)	(0.680–1.370)	(1.056–1.117)	(0.973–1.105)	
No tobacco usage							
Odds Ratio [SE]	0.913 [0.046]	0.986 [0.053]	0.680 [0.167]	0.830 [0.224]	0.930 [0.047]	0.999 [0.055]	−0.675
(95% CI)	(0.827–1.007)	(0.886–1.096)	(0.420–1.100)	(0.488–1.409)	(0.842–1.028)	(0.898–1.112)	
No alcohol usage							
Odds Ratio [SE]	0.773 [0.146]	0.901 [0.170]	0.818 [0.862]	1.297 [1.191]	0.764 [0.143]	0.894 [0.170]	0.397
(95% CI)	(0.534–1.118)	(0.622–1.304)	(0.104–6.450)	(0.215–7.839)	(0.528–1.103)	(0.615–1.298)	
Has health access							
Odds Ratio [SE]	1.192 *** [0.037]	1.175 *** [0.039]	1.655 ** [0.273]	1.745 ** [0.302]	1.163 *** [0.037]	1.145 *** [0.038]	2.391 *
(95% CI)	(1.121–1.267)	(1.102–1.253)	(1.199–2.286)	(1.243–2.450)	(1.094–1.238)	(1.073–1.222)	
No comorbidity							
Odds Ratio [SE]	0.598 *** [0.019]	0.722 *** [0.024]	0.417 *** [0.075]	0.459 *** [0.085]	0.607 *** [0.019]	0.736 *** [0.024]	−2.511 *
(95% CI)	(0.562–0.637)	(0.677–0.769)	(0.293–0.592)	(0.320–0.660)	(0.571–0.647)	(0.690–0.785)	
Durability & Quality of Structure							
Odds Ratio [SE]	1.137 *** [0.019]	1.084 *** [0.022]	1.111 [0.145]	1.100 [0.138]	1.148 *** [0.019]	1.096 *** [0.023]	0.027
(95% CI)	(1.100–1.174)	(1.041–1.128)	(0.861–1.434)	(0.861–1.406)	(1.111–1.186)	(1.053–1.142)	
Access to Housing Services/Infrastructure							
Odds Ratio [SE]	1.073 *** [0.016]	1.025 [0.022]	1.030 [0.068]	1.044 [0.091]	1.063 *** [0.016]	1.009 [0.022]	0.373
(95% CI)	(1.042–1.105)	(0.983–1.069)	(0.904–1.174)	(0.880–1.238)	(1.032–1.095)	(0.967–1.053)	
Indoor Air Quality							
Odds Ratio [SE]	1.072 *** [0.016]	1.016 [0.018]	1.065 [0.104]	1.026 [0.108]	1.076 *** [0.016]	1.025 [0.019]	0.011
(95% CI)	(1.041–1.104)	(0.980–1.052)	(0.879–1.289)	(0.835–1.262)	(1.044–1.108)	(0.989–1.062)	
Crowding							
Odds Ratio [SE]	1.181 *** [0.018]	1.096 *** [0.018]	0.857 [0.072]	0.782 ** [0.074]	1.187 *** [0.018]	1.103 *** [0.018]	−3.598 ***
(95% CI)	(1.147–1.216)	(1.061–1.132)	(0.728–1.009)	(0.650–0.940)	(1.152–1.223)	(1.068–1.140)	
Household Possessions							
Odds Ratio [SE]	1.003 [0.015]	0.895 *** [0.022]	1.007 [0.084]	0.986 [0.141]	0.995 [0.015]	0.886 *** [0.022]	0.740
(95% CI)	(0.974–1.033)	(0.852–0.940)	(0.855–1.186)	(0.746–1.304)	(0.965–1.025)	(0.844–0.931)	
Tenure security							
Odds Ratio [SE]	0.928 *** [0.014]	0.930 *** [0.014]	1.047 [0.080]	1.030 [0.084]	0.918 *** [0.014]	0.922 *** [0.014]	1.336
(95% CI)	(0.902–0.955)	(0.902–0.958)	(0.902–1.215)	(0.879–1.208)	(0.892–0.946)	(0.895–0.951)	
Intercept							
Odds Ratio [SE]		0.025 *** [0.006]		0.020 ** [0.025]		0.025 *** [0.006]	
(95% CI)		(0.015–0.041)		(0.002–0.223)		(0.015–0.041)	
N		68,422		4333		64,089	

Note: Crude ORs represent unadjusted bivariate associations calculated from cross-tabulations, while adjusted ORs are derived from multivariate logistic regression models controlling for socio-demographic and health-related covariates. Column (7) reports the *z*-statistics testing whether the coefficients of interest differ significantly between the slum and non-slum samples. * Significant at *p* < 0.05; ** significant at *p* < 0.01; *** significant at *p* < 0.001.

## Data Availability

The datasets analyzed during the current study are publicly available in the DHS Program repository at https://dhsprogram.com/data/dataset_admin/login_main.cfm (accessed on 17 September 2024).

## Supplementary Materials

The following supporting information can be downloaded at: https://www.mdpi.com/article/10.3390/ijerph22121817/s1, File S1: Detailed overview of study variables.

## Abbreviations

The following abbreviations are used in this manuscript:

LMICs	Low- and middle-income countries
HTN	Hypertension
SBP	Systolic blood pressure
DBP	Diastolic blood pressure
HICs	High-income countries
CARDIA	Coronary Artery Risk Development in Young Adults
NFHS-4	National Family Health Survey Wave 4
DHS	Demographic and Health Surveys
WHO	World Health Organization
IHCI	India Hypertension Control Initiative
SDoH	Social determinants of health
AORs	Adjusted odds ratios
CEBs	Census enumeration blocks
PSUs	primary sampling units
