# Supplementary material for: Urban Housing and Hypertension Among Women in India: Comparing Slum and Non-Slum Contexts Using National Survey Data
Source: Int J Environ Res Public Health. 2025 Dec 4;22(12):1817. doi: 10.3390/ijerph22121817 (PMC12732635; doi:10.3390/ijerph22121817)
Supplement: Supplementary file 1 [file ijerph-22-01817-s001.zip › ijerph-4002816-supplementary.pdf]

Supplementary File S1 Detailed overview of study variables

Detailed overview of study variables:

a. Demographic control variables:
   Socio-demographic variables were included as a control variables in the study, including, the respondent's current age as measured in years; highest educational level with multiple categories: no education, primary, secondary, and higher education; current marital status: never married, currently married, married, guana not performed, widowed, divorced, separated, deserted; wealth index: poorest, poorer, middle, richer, and richest; state including Madhya Pradesh, Maharashtra, Delhi, Tamil Nadu, Uttar Pradesh, West Bengal, and Telangana.

b. Health-related control variables:
   Tobacco Usage: Tobacco use was determined using responses from two NFHS questions indicating whether individuals had smoked or used any tobacco products, or those who used tobacco within 30 minutes prior to having their blood pressure measured.

   Alcohol Usage: This variable indicates whether respondents reported consuming any alcoholic beverages.

   Healthcare Access: The NFHS-4 survey included a series of questions addressing whether women experienced any barriers while seeking healthcare for themselves. These questions included difficulty in obtaining permission to seek care, financial resources, distance to health facility, transportation, going alone, availability of healthcare providers, availability of female providers, and availability of necessary medications. Individuals who reported a "big problem" for any of these items were classified as having no healthcare access; otherwise, they were considered to have healthcare access.

   Comorbidity: Self-reported data on five health conditions is available in NFHS-4 data including diabetes, asthma, thyroid disease, heart disease, and cancer. In addition, a BMI category variable was incorporated to identify individuals who were underweight (BMI < 18.5) or overweight (BMI ≥ 25) according to WHO guidelines, which are commonly employed in public health research on hypertension risk factors within Asia. Respondents who responded "Yes" to having any of these conditions or were under/overweight were identified as having comorbidities.

c. Outcome variable: Hypertension
   Hypertension in this study is defined as either self-reported diagnosis or undiagnosed. Self-reported hypertension refers to respondents who answered "Yes" when asked if a doctor had diagnosed them with high blood pressure. Undiagnosed hypertension is determined by average systolic blood pressure readings $\geq$140 mmHg or diastolic blood pressure readings

≥90 mmHg based on the survey's biomarker data, following Indian guidelines and conventions in related literature. The NFHS-4 survey utilized an electronic sphygmomanometer (Omron Blood Pressure Monitor) to record systolic and diastolic blood pressure measurements for all participants.

d. Predictor Variable: Housing Quality
A composite housing quality construct was developed to reflect the multi-dimensional nature of housing quality conditions, drawing on six core domains from the NFHS-4 dataset: (1) durability and quality of structure, (2) access to essential services or infrastructure (e.g., water, sanitation, electricity), (3) household possessions (basic assets), (4) indoor air quality (ventilation, cooking fuel), (5) crowding (number of individuals per room), and (6) tenure security (legal recognition of property rights). The details of how each domain is constructed are:

1. Durability and Quality of Structure
    a. House Type (htscore): Derived from variable shnfhs2, indicating the overall type or classification of the dwelling.
    b. Main Floor Material (mfmscore): Based on hv213, with higher scores for higher-quality floor materials (e.g., cement or tiled floors).
    c. Main Wall Material (mwmscore): Based on hv214, scoring different wall materials for durability and protection.
    d. Main Roof Material (mrmscore): Based on hv215, reflecting roof construction that offers greater durability and protection from weather.
    After each item is scored (0, 1, or 2), the variables are standardized and averaged to create a domain-specific housing quality score, which is then standardized again for comparability between the dimensions.

2. Access to Services/Infrastructure
    a. Water Source (wsscore): Derived from hv201, differentiating among piped, improved, or unimproved water sources.
    b. Time to Get Water (twscore): Based on hv204, capturing whether water is readily available (e.g., 0 minutes) or requires significant travel time.
    c. Location of Water Source (lswscore): From hv235, indicating whether water is available on-premises, nearby, or farther away.
    d. Type of Toilet Facility (tfscore): Based on hv205, scoring flush toilets, pit latrines, or open defecation.
    e. Shared Toilet (stscore and nstscore): From hv225 and hv238, indicating whether a toilet is shared with other households and, if so, how many.
    f. Electricity (elecscore): From hv206, reflecting the presence (score = 2) or absence (score = 0) of electricity in the household.

g. Place to Wash Hands (whscore): Based on hv230b, scoring whether a designated handwashing facility is present.
   All these items are scored (0, 1, or 2), standardized individually, and averaged into a standardized “access to services/infrastructure” score

3. Crowding
   a. Crowding is measured by number of people per room, calculated by dividing the total number of household members (v136) by the number of rooms (hv216). A three-level score assigns 0 for severe crowding (>4 people/room), 1 for moderate crowding (≥3 people/room), and 2 for minimal crowding (≤2 people/room). This crowding score is also standardized.

4. Indoor Air Quality
   a. Cooking Fuel (cfscore): Based on hv226, distinguishing clean fuels (e.g., electricity, LPG) from less clean fuels (wood, dung, etc.).
   b. Food Cooked Over (scoscore): From sh39, indicating whether food is cooked over an open fire, stove, or other method.
   c. Food Cooked Where (hsoscore): Based on hv241, indicating whether cooking is done indoors, outdoors, or in another enclosed space.
   d. Separate Kitchen (skscore): From hv242, scoring whether the household has a separate kitchen area.
   After scoring each variable, the sub-scores are averaged and standardized to compute the indoor air quality score.

5. Household Possessions
   a. Includes household asset indicators: mattress (matscore), personal computer (pcscore), chair (chrscore), bed (bedscore), table (tbscore), electric fan (efscore), radio (rdscore), bicycle or two-wheeler (bwtscore), car (ctscore), refrigerator (rftscore), cooler (clscore), and washing machine (wmscore). Each asset is coded as 2 if present and 0 if absent, then aggregated into a single comfort score, standardized as needed.

6. Tenure Security (score_tenure)
   a. Own House (ohscore): From sh46, indicating whether the respondent reports ownership of the dwelling (score = 2) or not (score = 0). This is then incorporated into a tenure security score and standardized.

Indicators within each domain were scored on a scale of 0 to 2, where 0 represents poor quality, 1 indicates acceptable quality, and 2 corresponds to good quality. Sub-scale scores were standardized and averaged to create dimension-specific scores, which were

further standardized to ensure comparability across all domains. A higher score indicates better overall housing quality. This approach allows for a more holistic assessment of the living environment, recognizing the interplay between physical infrastructure, resource availability, and conditions that may affect health outcomes.

Table S1. Descriptive analysis of non-hypertensive and hypertensive women in seven Indian states, NFHS-4, 2015-2016.

	Hypertension		
	Non- Hypertensive	Hypertensive	Test
N	65440 (85.7%)	10948 (14.3%)	
**Age**			<0.001
Mean (SD)	29.263 (9.669)	36.466 (8.813)	
Median (Q1,Q3)	28 (21, 37)	38 (30, 44)	
Min-Max	(18-49)	(18-49)	
**Educational level**			
no education	11454 (17.5%)	2,398 (21.9%)	<0.001
primary	7166 (11.0%)	1,455 (13.3%)	
secondary	32600 (49.8%)	5,083 (46.4%)	
higher	14220 (21.7%)	2,012 (18.4%)	
**Marital status**			
never married	20392 (31.2%)	924 (8.4%)	<0.001
currently married	42,339 (64.7%)	9,311 (85.0%)	
married, gauna not performed	60 (0.1%)	3 (0.0%)	
widowed	1,862 (2.8%)	539 (4.9%)	
divorced	277 (0.4%)	64 (0.6%)	
separated	398 (0.6%)	92 (0.8%)	
deserted	112 (0.2%)	15 (0.1%)	
**Wealth index**			
Mean (SD)	4.005 (1.088)	4.102 (0.997)	<0.001
Median (Q1,Q3)	4 (3, 5)	4 (4, 5)	
Min-Max	(1-5)	(1-5)	
**State**			
Madhya Pradesh	15,844 (24.2%)	1,858 (17.0%)	<0.001
Maharashtra	9,111 (13.9%)	1,065 (9.7%)	
Delhi	3,951 (6.0%)	514 (4.7%)	
Tamil Nadu	8,697 (13.3%)	3,506 (32.0%)	
Uttar pradesh	22,022 (33.7%)	2,949 (26.9%)	
West Bengal	3,898 (6.0%)	599 (5.5%)	
Telangana	1,917 (2.9%)	457 (4.2%)	
**Hypertension Prevalence**			
Has Hypertension			
**Average Systolic**			<0.001
Mean (SD)	111.399 (11.013)	124.926 (18.617)	
Median (Q1,Q3)	111 (103.5, 119)	122.333 (110, 141)	
Min-Max	(66 -139.667)	(69.333-169)	
**Average Diastolic**			<0.001
Mean (SD)	75.136 (7.379)	83.849 (12.222)	
Median (Q1,Q3)	75.667 (70.000, 80.667)	82.000 (75.667, 90.000)	
Min-Max	(42.333-89.667)	(46.000-146.000)	
**Comorbidity**			

		Hypertension	
Yes	31,224 (47.7%)	6,824 (62.3%)	<0.001
**Healthcare Access**			
Has Health Access	39,584 (60.5%)	6,262 (57.2%)	<0.001
**Tobacco Usage**			
Yes	3,093 (4.7%)	658 (6.0%)	<0.001
**Alcohol Usage**			
Yes	268 (0.4%)	64 (0.6%)	0.010
**Durability and Structural Quality**			<0.001
Mean (SD)	-0.018 (1.011)	0.109 (0.922)	
Median (Q1,Q3)	0.485 (-0.126, 0.485)	0.485 (-0.072, 0.485)	
Min-Max	(-3.895-1.096)	(-3.895-1.096)	
**Access to Housing Services & Infrastructure**			0.004
Mean (SD)	-0.004 (1.011)	0.026 (0.928)	
Median (Q1,Q3)	0.257 (-0.372, 0.948)	0.257 (-0.372, 0.551)	
Min-Max	(-6.013-1.127)	(-5.502-1.127)	
**Indoor Air Quality**			<0.001
Mean (SD)	-0.011 (1.002)	0.066 (0.985)	
Median (Q1,Q3)	0.730 (-0.345, 0.730)	0.730 (-0.345, 0.730)	
Min-Max	(-2.875-0.779)	(-2.875-0.779)	
**Crowding**			<0.001
Mean (SD)	-0.028 (0.989)	0.168 (1.046)	
Median (Q1,Q3)	-0.805 (-0.805, 1.463)	-0.805 (-0.805, 1.463)	
Min-Max	(-0.805-1.463)	(-0.805-1.463)	
**Household Possessions**			0.953
Mean (SD)	0.000 (1.003)	-0.001 (0.985)	
Median (Q1,Q3)	-0.204 (-0.204, 1.265)	-0.204 (-0.204, 1.265)	
Min-Max	(-1.672-1.265)	(-1.672-1.265)	
**Tenure security**			<0.001
Mean (SD)	0.009 (0.994)	-0.055 (1.033)	
Median (Q1,Q3)	0.550 (0.550, 0.550)	0.550 (-1.817, 0.550)	
Min-Max	(-1.817-0.550)	(-1.817-0.550)	
